# Complement Activation May Drive the Pathogenicity of Anti-α6 and Anti-β4 Integrin Antibodies In Vivo

**DOI:** 10.3390/biom16030417

**Published:** 2026-03-12

**Authors:** Gefei Du, Shirin Emtenani, Dennis Niese, Jian Liu, Ferdinand Gebauer, Neele J. Dunst, Aysun Gökce, Kristina Spaniol, Florian Groeber-Becker, Jelena Šimunović, Mislav Novokmet, Gerd Geerling, Kyle T. Amber, Markus H. Hoffmann, Ralf J. Ludwig, Katja Bieber, Stephanie Goletz, Gang Zhou, Enno Schmidt, Sabrina Patzelt

**Affiliations:** 1 Lübeck Institute of Experimental Dermatology, University of Lübeck, 23562 Lübeck, Germany; dugefei18@whu.edu.cn (G.D.); dennis.niese@uksh.de (D.N.); jian.liu@uksh.de (J.L.); ferdinand.gebauer@student.uni-luebeck.de (F.G.); n.dunst@student.uni-luebeck.de (N.J.D.); aysun.gokce95@gmail.com (A.G.); ralf.ludwig@uksh.de (R.J.L.); katja.bieber@uksh.de (K.B.); stephanie.goletz@uksh.de (S.G.); enno.schmidt@uksh.de (E.S.); sabrina.patzelt@uksh.de (S.P.); 2 State Key Laboratory of Oral & Maxillofacial Reconstruction and Regeneration, Key Laboratory of Oral Biomedicine Ministry of Education, Hubei Key Laboratory of Stomatology, School & Hospital of Stomatology, Wuhan University, Wuhan 430079, China; zhougang@whu.edu.cn; 3 Department of Oral Medicine, School and Hospital of Stomatology, Wuhan University, Wuhan 430079, China; 4 Department of Ophthalmology, University Clinic Düsseldorf, 40225 Düsseldorf, Germany; kristinaspaniol@yahoo.de (K.S.); florian.groeber-becker@isc.fraunhofer.de (F.G.-B.); geerling@med.uni-duesseldorf.de (G.G.); 5 Translational Center for Regenerative Therapies (TLC-RT), Fraunhofer Institute for Silicate Research (ISC) Würzburg, 97082 Bavaria, Germany; 6 Genos Glycoscience Research Laboratory, 10000 Zagreb, Croatia; jsimunovic@genos.hr (J.Š.); mislav.novokmet@selvita.com (M.N.); 7 Department of Dermatology, Rush University Medical Center, Chicago, IL 60612, USA; kyle_amber@rush.edu; 8 Department of Internal Medicine, Rush University Medical Center, Chicago, IL 60612, USA; 9 Institute for Systemic Inflammation Research, University of Lübeck, 23562 Lübeck, Germany; markus.hoffmann@uni-luebeck.de; 10 Department of Dermatology, Allergology and Venerology, University of Lübeck, 23562 Lübeck, Germany

**Keywords:** mucous membrane pemphigoid, autoantibody, mouse model, α6/β4 integrin, CXCL2, complement

## Abstract

Autoantibodies targeting α6β4 integrin have been identified in individual patients with mucous membrane pemphigoid (MMP). Reactivity against α6 integrin has been associated with oral lesions, while anti-β4 integrin reactivity has been linked to ocular involvement. However, the pathogenic effects of these antibodies have not been fully elucidated. Here, we investigated the pathogenic potential of anti-α6 and anti-β4 integrin IgG both in vitro and in vivo. Immune complexes of anti-α6 and anti-β4 integrin induced the release of reactive oxygen species from normal human leukocytes and stimulated CXCL2 secretion in cultured murine C5N keratinocytes. In vivo, repeated injections of IgG against a recombinant fragment of β4 integrin into C57BL/6 mice led to palpebral conjunctival swelling and mild oral lesions. The latter was observed following injection of IgG against a recombinant fragment of α6 integrin. Histopathological analysis revealed subepithelial inflammatory infiltrates without evidence of split formation. Direct immunofluorescence microscopy showed linear deposits of IgG at the basement membrane zone in most tissues, whereas C3 deposition was largely absent. This lack of complement activation was corroborated by a complement fixation assay, which confirmed that IgG against α6 and β4 integrin failed to induce C3 deposition in normal murine conjunctivae, buccal mucosa, or skin. Collectively, these findings indicate that IgG autoantibodies against α6 and β4 integrin exhibit pathogenic activity in vitro and induce mild disease in vivo, possibly due in part to relatively inefficient complement activation in this model.

## 1. Introduction

Mucous membrane pemphigoid (MMP) is a mucocutaneous autoimmune blistering disorder characterized by autoantibodies targeting structural proteins of the basement membrane zone (BMZ), with predominant mucosal involvement [1]. The most common target antigens include BP180 (type XVII collagen) and laminin 332, while approximately 5% of patients develop autoantibodies against type VII collagen. Additionally, reactivity against α6β4 integrin has been described in a subset of patients, and anti-BP230 antibodies are detected in a minority of cases, nearly always in combination with anti-BP180 reactivity [2,3]. Clinically, MMP patients present with erosions affecting surface-close mucous membranes, most frequently the oral cavity and conjunctiva, but may also involve the nasal, pharyngeal, and genital mucosa. The disease carries significant potential for scarring and profound morbidity [3]. Severe complications, including visual impairment or blindness, as well as esophageal or tracheal strictures, can drastically impact a patient’s quality of life [4]. Moreover, an association between anti-laminin 332 MMP and solid malignancies has been reported [5,6,7]. When disease manifestations are restricted to a single anatomical site, the terms ocular MMP and oral MMP are recommended to specify disease localization [3,8].

Previous studies have suggested that anti-α6 integrin autoantibodies are preferentially associated with oral lesions, while anti-β4 integrin has been linked to ocular involvement in MMP [9,10]. However, the prevalence of these autoantibodies among MMP patients remains largely unexplored [11,12,13,14]. The pathogenic potential of anti-α6β4 integrin antibodies has been demonstrated in vitro using cultured human bulbar conjunctiva [15] and normal human buccal mucosa [16,17]. Although these in vitro studies showed binding to skin, oral, and conjunctival tissues, in vivo experiments in neonatal mice injected with anti-β4 integrin antibodies primarily induced subepidermal blistering of the skin, without replicating oral or ocular lesions characteristic of MMP [12].

In the present study, we aimed to establish a mouse model of MMP by repeated injections of rabbit anti-mouse IgG directed against the α6 or β4 integrin into adult C57BL/6 mice. Treated mice developed mild macroscopic and microscopic mucosal lesions without obvious skin involvement attributable to anti-α6 or anti-β4 integrin antibody injections. The limited tissue damage may reflect relatively inefficient complement activation at the skin or mucosal tissues in this model.

## 2. Materials and Methods

### 2.1. Cloning and Expression of Murine α6 and β4 Integrin Fragments

Fragments of murine α6 integrin (amino acids 217–590; UniProt ID Q61739) and β4 integrin (amino acids 1485–1817; UniProt ID A2A863) were synthesized using the GeneArt service (Thermo Fisher Scientific, Waltham, MA, USA). The selected sequences corresponded to the primary epitopes of the human proteins described by Rashid et al. [11] and Bhol et al. [13]. Previous studies using sera from MMP patients without ocular involvement and ocular pemphigoid revealed reactivity against two peptides (residues 1489–1510 and 1689–1702) in the intracellular domain of β4 integrin [12]. To ensure that both peptide sequences were encoded, a longer sequence (residues 1489–1822) was selected. In contrast, sera from MMP patients with ocular involvement primarily reacted with a single peptide (residues 292–305) in the extracellular domain of α6 integrin [11], for which a longer sequence (residues 217–501) was also used. The corresponding murine fragments were expressed in E. coli Rosetta (DE3) cells (Novogene, Martinsried, Germany) as His-tag fusion proteins and purified using immobilized metal affinity chromatography. Protein concentration was determined using a BSA standard curve (0.65–2 mg/mL) on a PageBlue (Thermo Fisher Scientific) stained SDS-PAGE gel.

### 2.2. Generation and Characterization of Rabbit Anti-Mouse IgG Against Integrin α6 and β4 Subunits

To generate antibodies against murine α6 and β4 integrin subunits, rabbits were immunized with recombinant α6 or β4 integrin fragments (Eurogentec, Seraing, Belgium). Total IgG was purified from rabbit serum using Protein G agarose. Antigen-specific IgG was subsequently purified against murine integrin a6/β4 subunits. This was achieved by affinity chromatography using Sepharose beads (GE Healthcare, Düsseldorf, Germany) coupled with the respective recombinant proteins, following previously established protocols [18,19,20]. The concentration of rabbit IgG was determined spectrophotometrically at 280 nm.

### 2.3. ROS Release Assay

The assay was performed to quantify reactive oxygen species (ROS) release from immobilized immune complexes (IC) following incubation with normal human polymorphonuclear leukocytes (PMNs), as previously described [21]. In short, high-binding 96-well white microtiter plates (Greiner BioOne, Frickenhausen, Germany) were coated overnight at 4 °C with recombinant α6 or β4 integrin proteins (10, 20, 40, or 80 µg/mL in urea 6 M) while shaking. After incubation, the plates were washed with PBS containing 0.5% Tween 20 (PBS-T) and blocked with 5% skimmed milk in PBS-T for 1 h at 37 °C. Following additional washes, wells were incubated for 1 h at room temperature (RT) with rabbit anti-α6 integrin IgG or anti-β4 integrin IgG (10, 20, 40, or 80 µg/mL in blocking buffer) to allow IC formation. Meanwhile, PMNs were isolated from healthy donors (*n* = 3) using PolymorphPrep™ (Axis-Shield, Heidelberg, Germany) according to the manufacturer’s protocol. The purified PMNs were resuspended in a chemiluminescence medium (color-free RPMI-1640; Sigma-Aldrich, Munich, Germany) supplemented with 1% heat-inactivated fetal bovine serum, 2 g/L glucose, and 25 mM HEPES containing 1 mM luminol (Sigma-Aldrich). A total of 2 × 10^5^ PMNs were seeded into each well, and the chemiluminescence reaction was monitored over 2 h at 37 °C using a GloMax^®^ Discover System microplate reader (Promega, Mannheim, Germany). Statistical significance was assessed using the Kruskal–Wallis test followed by Dunn’s multiple comparisons test.

### 2.4. Cryosection Assay

To evaluate the blister-inducing potential of anti-murine α6 and β4 integrin antibodies, the cryosection model, an established ex vivo model of immune complex-induced dermal-epidermal separation, was applied, as previously described [22,23,24,25]. In brief, cryosections of murine skin were incubated with 30 µL of purified anti-α6 integrin IgG or anti-β4 integrin IgG (1 mg) for 1 h at 37 °C. Anti-mCOL7 IgG (targeting the immunodominant domain of type VII collagen; 1 mg/mL) served as a positive control, and normal rabbit IgG (NR IgG; 1 mg/mL) served as a negative control. Leukocytes were isolated from healthy blood donors via dextran 500 (Roth, Karlsruhe, Germany) sedimentation. Following PBS washing, the isolated leukocytes were incubated with the skin sections for 3 h at 37 °C. After incubation, most leukocytes were removed by washing with PBS. Skin sections were then stained with haematoxylin and eosin (H&E) and examined at 200× magnification using a Keyence microscope (BZ-9000E Series, Keyence GmbH, Neu-Isenburg, Germany). The experiment was performed in three independent repetitions using three different blood donors as well as different mouse skin sections.

### 2.5. Cell Culture Experiments

C5N murine keratinocyte cells (a kind gift from Dr. Allan Balmain, UCSF, USA, with the help of distribution from Dr. Christina Seebode, University of Rostock, Germany) were used for cell culture experiments [26,27]. Cells were seeded on chamber slides at a density of 5 × 10^5^ cells per chamber or per well in 24-well plates. The cells were allowed to adhere and grow under standard culture conditions (37 °C, 5% CO_2_, humidified atmosphere). After approximately 80–90% confluence was reached, cells were washed three times with PBS. For IF staining, cells on chamber slides were incubated with 200 µg of anti-α6 or anti-β4 integrin IgG for 4 h, washed, fixed with 4% PFA, washed again, and permeabilized with 0.5% Triton for 10 min at 4 °C. After additional washing, anti-rabbit IgG FITC at 1:100 dilution (Jackson Immuno Research, West Grove, PA, USA) was added for one h at 37 °C. Cell nuclei were counterstained with DAPI (Sigma-Aldrich) after washing. Fluorescence imaging was performed using a Keyence fluorescence microscope. For cytokine release experiments, cells were co-incubated with anti-α6 or anti-β4 integrin IgG at concentrations of 2.5, 5, and 10 mg or 10 mg of NR IgG. After one day, supernatants were collected and removed from cells and debris by centrifugation. Supernatants were analyzed for the presence of CXCL2 using ELISA, following the manufacturer’s instructions (R&D Systems).

### 2.6. Western Blot

Keratinocyte cell lysate was prepared from one full 175 cm^2^ cell culture flask of murine C5N cells by lysing the cells in 1 mL of 5 × SDS Laemmli buffer. A total of 150 µL of lysate was separated on a standard SDS-polyacrylamide gel and transferred onto nitrocellulose membranes (GE Healthcare). Membranes were blocked with 5% milk powder in Tris-buffered saline containing 0.1% Tween-20 (TBS-T) for 1 h at RT. After three washes with TBS-T1 the following primary antibodies and sera were diluted in TBS-T1 containing 1% BSA for detection: goat anti-mouse β4 integrin HRP, 1:2000 (R&D Systems, Minneapolis, MN, USA), rabbit anti-α6 integrin HRP, clone EPR18124, 1 µg/mL (abcam, Cambridge, UK), serum samples from rabbits immunized with α6/β4 integrin rabbits, 1:40,000, as well as pre-immune serum from these rabbits, 1:10,000, isolated total IgG from rabbits immunized with murine α6/β4 integrin, 1:100,000, specifically purified α6/β4 integrin IgG, 1:2000. Blots were incubated with primary antibody overnight at 4 °C, washed three times in TBS-T1, and incubated with secondary antibody for 1 h at RT. Secondary antibodies corresponding to the species of the primary antibody: goat anti-rabbit HRP 1:1000, rabbit anti-goat (Agilent Technologies, Glostrup, Denmark) 1:500. Protein detection was performed by enhanced chemiluminescence (ECL, Thermo Fisher, Thermo Fisher Scientific, Dreieich, Germany) following the manufacturer’s instructions and detection using ChemiDoc Imaging System (Bio-Rad, Hercules, CA, USA).

### 2.7. Antibody-Transfer Mouse Model of MMP

All animal experiments were approved by the Ministry of Agriculture, Rural Areas, Europe, and Consumer Protection of Schleswig-Holstein and conducted in accordance with EULAR guidelines by certified researchers. Adult C57BL/6J mice were purchased from Jackson Laboratory (Bar Harbor, ME, USA) and housed under specific pathogen-free conditions in the animal facility of the University of Lübeck. The mice were maintained on a 12 h light-dark cycle with unrestricted access to food and water. Antibody injections and clinical examinations were performed under anesthesia, achieved through intraperitoneal (i.p.) administration of ketamine (100 µg/g body weight) and xylazine (15 µg/g body weight). The antibody transfer anti-laminin 332 MMP mouse model was previously described [20]. Briefly, IgG against the α3 chain of murine laminin 332 (mLAMA3) was isolated from sera of New Zealand rabbits immunized with the middle and C-terminal regions of the mLAMA3 α3 chain (Eurogentec, Seraing, Belgium) using protein G sepharose (Genscript, Piscataway, NJ, USA). Mice received subcutaneous injections of 6 mg anti-mLAMA3 IgG at the neck every other day from day 0 to day 10. All experiments were conducted in age- and sex-matched mice, 8–12 weeks old. Clinical scoring followed a well-established scoring system. Mice were sacrificed via cervical dislocation under anesthesia, and tissue biopsies were subsequently taken for further investigations [18]. Endoscopic examinations were performed using a high-resolution small animal endoscope (Karl Storz AidaVet, Tuttlingen, Germany) as previously described [28].

### 2.8. Immunofluorescence and Histology

Frozen sections of 6 µm thickness of mouse peri-injectional skin, buccal mucosa, and palpebral conjunctival tissues were investigated by direct immunofluorescence (IF) using FITC-conjugated anti-rabbit IgG (Jackson Immuno Research, West Grove, PA, USA) and anti-mouse C3 (MP Biomedicals, Solon, OH, USA) antibodies diluted at 1:100 and 1:50, respectively. Sections that omitted the FITC-conjugated IgG were used as negative controls. For histopathology, 4.5 µm paraffine sections were haematoxylin and eosin (H&E) stained.

### 2.9. Complement Fixation Aassay

The complement-fixing ability of anti-integrin IgG antibodies was evaluated using a modified complement fixation test [29]. Cryosections of healthy mouse skin, buccal mucosa, and conjunctiva were incubated with heat-inactivated anti-α6 or anti-β4 integrin IgG (10 mg per section) in PBS containing 1% BSA for 1 h at 37 °C. After washing with PBS-T, the sections were subjected to pooled fresh human hirudin plasma, serving as a complement source, for 1.5 h at 37 °C. Subsequently, the sections were stained with FITC-conjugated anti-human complement C3 IgG (dilution 1:100; MP Biomedicals, Eschwege, Germany) for 1 h at 37 °C. After staining, slides were washed and mounted with DAPI Fluoromount G (Southern Biotech, Birmingham, AL, USA). Anti-Col7 IgG and NR IgG were used as positive and negative controls, respectively. C3 deposition along the BMZ was evaluated semi-quantitatively using a Keyence microscope.

### 2.10. Glycosylation Analysis

N-glycans from 200 µg of IgG per sample (NR, anti-α6, anti-β4, and LAMA3) were released according to the previously reported protocol for IgG deglycosylation [30]. Briefly, IgG was dried in a vacuum concentrator and dissolved in 30 µL of 1.33% sodium dodecyl sulphate (SDS) and denatured at 65 °C for 10 min. After cooling down to RT, 10 µL of 4% Igepal-CA630 (Sigma-Aldrich) was added to the samples to neutralize the inhibitory effect of SDS on PNGase F (Promega, Madison, WI, USA) activity. PNGaseF (24 mU/µL) in 5× PBS was added to the samples and incubated overnight at 37 °C to release N-glycans.

N-glycans were labeled with procainamide (ProA, 43.2 mg/mL, Sigma-Aldrich) in a 25 µL mixture of dimethyl sulfoxide (DMSO) and glacial acetic acid (70:30, *v*/*v*), using 2-methylpiridine borane complex (44.8 mg/mL, Sigma-Aldrich) as a reducing agent in two steps. First, 25 µL of the mixture with the label was added and incubated at 65 °C for 60 min, and afterwards 25 µL of the mixture with the reducing agent was added, and the incubation was continued for another 90 min [31]. After labelling, IgG N-glycans were purified by hydrophilic interaction chromatography–solid-phase extraction (HILIC–SPE) on a 0.2 µm pore size AcroPrep wwPTFE filter plate (Pall Corporation, New York, NY, USA) according to the protocol published before and eluted with ultra-pure water into a PCR plate (Thermo Fisher Scientific, Waltham, MA, USA) [32]. Analysis was performed using a Waters Acquity H-class UHPLC system and ProA-labeled (excitation wavelength 310 nm, emission wavelength 370 nm). IgG N glycans were separated into 31 chromatographic peaks on a Waters bridged ethylene hybrid (BEH) glycan chromatographic column (100 mm × 2.1 mm, 1.7 µm BEH particles) maintained at 60 °C. The separation was performed in a 35 min analytical run at a flow rate of 0.4 mL/min with a gradient of 75–62% solvent B (ACN), while solvent A was 100 mM ammonium formate, pH 4.4. Chromatograms were processed by Empower 3 software (Waters, Milford, MA, USA) and manually integrated. Each peak was expressed as a percentage of the total normalized area.

Structures in the peak were analysed and determined by UHPLC coupled to ESI-qTOF-MS (Bruker Daltonics, Bremen, Germany). Mass spectra were recorded in the range from 50 to 2750 *m*/*z* with a frequency of 0.5 Hz. The three precursors with the highest intensities were automatically selected for CID fragmentation. Features detected in MS spectra, created as a sum spectrum for a specific chromatographic peak, were searched for possible ProA-labeled N-glycan composition in GlycoMod, ExPASy tool (ExPASy, SIB Swiss Institute of Bioinformatics, https://web.expasy.org/glycomod/, accessed 17 January 2025), based on a singly charged *m*/*z* [33]. MS/MS spectra were searched for one of the following diagnostic ions in order to confirm N-glycan fragmentation: 204.0867 *m*/*z* for Hexose (H), 366.1385 *m*/*z* for N-acetylhexosamine (N), 441.3022 *m*/*z* for ProA linked to N, 673.2298 *m*/*z* for N-glycolylneuraminic acid (G) linked to H and N, 790.439 *m*/*z* for core F linked to two N and ProA. Recorded MS/MS spectra were also used to propose N-glycan structures in the chromatographic peak.

### 2.11. Statistics

All results are expressed as mean ± standard error of the mean (SEM). To assess affected body surface area over time, a two-way ANOVA followed by Holm–Sidak’s post hoc test was used. For analysis of endoscopic and conjunctival scores, the Kruskal–Wallis test with Dunn’s post hoc correction was applied. Statistical significance was defined as *p* < 0.05 for all analyses. Data processing and statistical analyses were conducted using GraphPad Prism, version 10 (GraphPad Software, San Diego, CA, USA).

## 3. Results

### 3.1. Generation and Characterization of Antibodies Against Murine α6 and β4 Integrin

**Figure 1:**
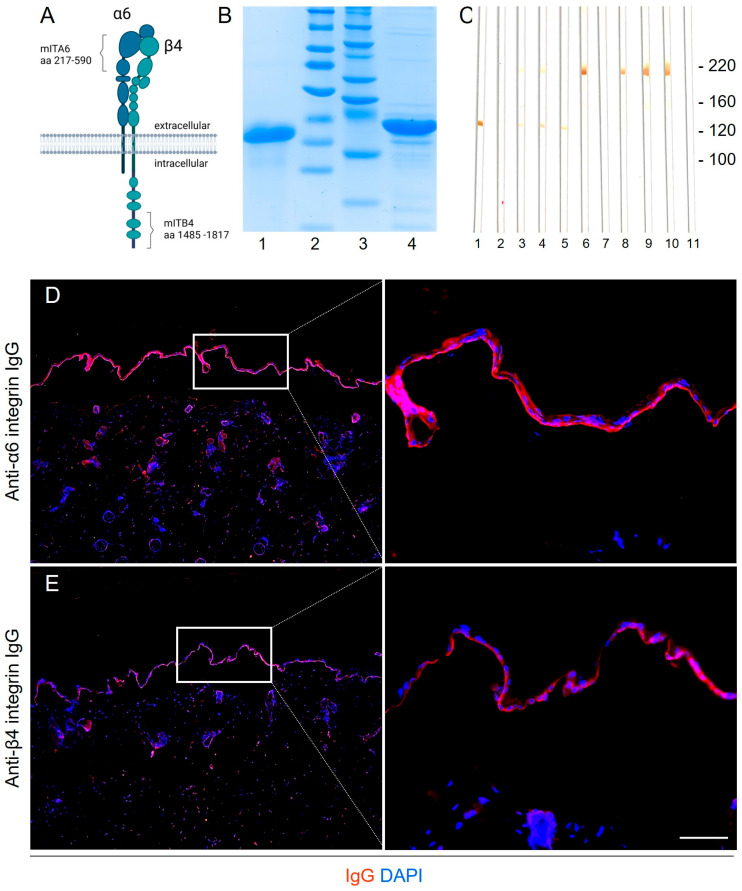
Generation and characterization of anti-α6 and anti-β4 integrin IgG. (**A**) Schematic representation of the murine α6β4 integrin heterodimer. (**B**) Recombinant fragments of murine α6 integrin (amino acids 217–590) and murine β4 integrin (amino acids 1485–1817) were expressed in E. coli and affinity-purified. SDS-PAGE gel with PageBlueTM staining shows α6 integrin (lane 1) and β4 integrin (lane 4) fragments at approximately 120 kDa and 200 kDa, respectively. Molecular weight markers are shown in lanes 2 and 3. (**C**) Immunoblot analysis using sera from rabbits immunized with α6 or β4 integrin fragments detected corresponding bands in murine C5N keratinocyte lysates at approximately 120 kDa (lane 3) and 200 kDa (lane 8), respectively. Affinity-purified total IgG (lanes 4 and 9) and specific IgG (lanes 5 and 10) showed bands with the same molecular weights. Affinity-purified anti-α6 (lane 1) and anti-β4 integrin IgG (lane 6) showed similar results, whereas pre-immune rabbit sera (lanes 2 and 7) and normal rabbit (NR) IgG (lane 11) showed no detectable signals. The molecular weight marker is indicated on the right. (**D**,**E**) Indirect immunofluorescence microscopy confirmed binding of (**D**) anti-α6 integrin IgG and (**E**) anti-β4 integrin IgG to the epidermal side of the salt-split murine skin. DAPI (blue) was used to stain the nuclei. Scale bar: 50 µm.

Rabbit antibodies were generated against fragments of murine α6 and β4 integrin subunits, homologous to fragments of human α6 and β4 integrin previously identified as immunodominant regions in MMP patients [11,12] (Figure 1A,B). Only sera with anti-BMZ titers exceeding 1:40,000, as determined by indirect IF on mouse tail tissue, were used for IgG purification. Purified anti-α6 and anti-β4 integrin IgG recognized bands at ~120 kDa and ~220 kDa, respectively, in murine keratinocyte cell lysates by Western blotting (Figure 1C). Additionally, indirect IF microscopy confirmed antibody binding to the epidermal side of salt-split mouse skin (Figure 1D,E).

### 3.2. Anti-α6 and Anti-β4 Integrin IgG Exhibit Pathogenic Potential In Vitro

**Figure 2:**
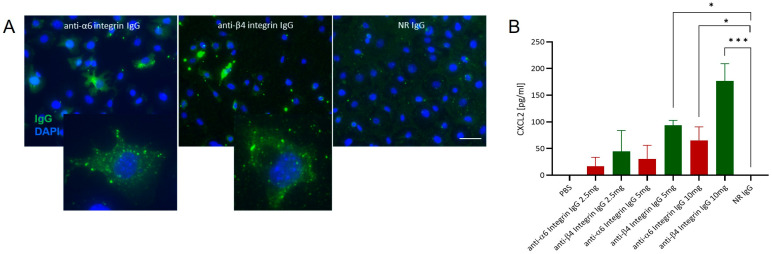
In vitro binding of anti-α6 and anti-β4 integrin IgG to cultured murine keratinocytes induces CXCL2 secretion. (**A**) Indirect immunofluorescence staining demonstrates binding of anti-α6 and anti-β4 integrin IgG (green) to the surface of murine keratinocytes. In contrast, normal rabbit (NR) IgG (negative control) shows no binding. Nuclei are counterstained with DAPI (blue). Scale bar: 50 µm. (**B**) ELISA analysis of CXCL2 levels in culture supernatants following 24 h incubation of murine keratinocytes with increasing concentrations (2.5, 5, and 10 µg/mL) of anti-α6 or anti-β4 integrin IgG. CXCL2 secretion increased in a dose-dependent manner, while neither PBS nor NR IgG controls triggered CXCL2 secretion. Data from five independent experiments were analyzed using one-way ANOVA and the Holm–Sidak multiple comparisons test. * *p* < 0.05; *** *p* < 0.001.

To assess the pathogenic potential of anti-α6 and anti-β4 integrin IgG, we utilized in vitro cultured murine C5N epidermal keratinocytes. Both antibody fractions specifically bound to C5N keratinocytes (Figure 2A) and led to a dose-dependent release of C-X-C motif chemokine ligand 2 (CXCL2) upon incubation with different concentrations of anti-murine α6 or β4 integrin IgG for 24 h. This effect was more pronounced with anti-β4 integrin IgG compared to anti-α6 integrin IgG. In contrast, no CXCL2 secretion was observed following incubation with NR IgG (Figure 2B).

**Figure 3:**
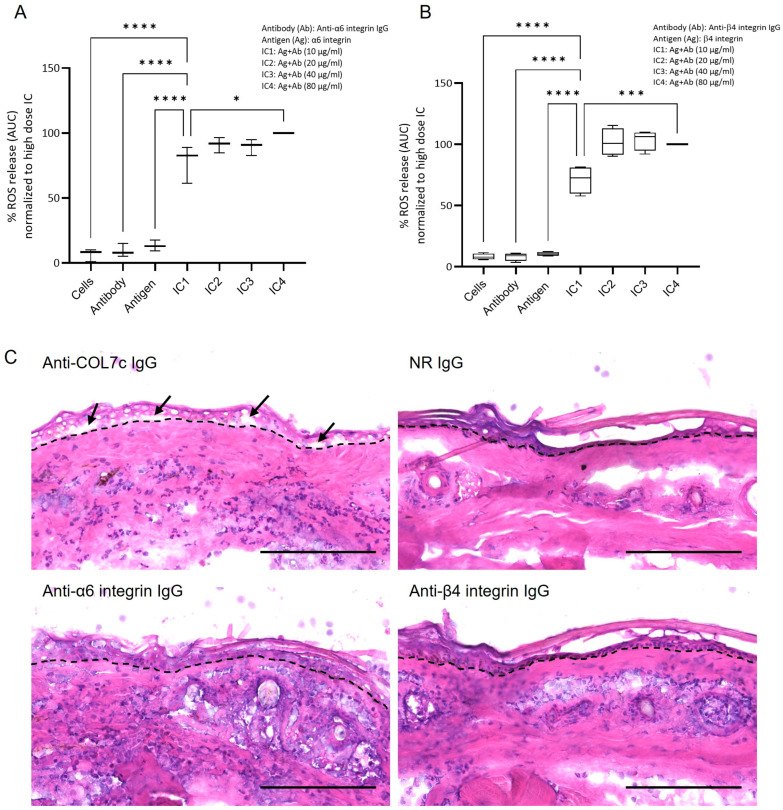
Pathogenicity of anti-α6 and anti-β4 integrin IgG in vitro and ex vivo. (**A**,**B**) The dose-dependent release of reactive oxygen species (ROS) from normal human polymorphonuclear leukocytes (PMNs) was assessed following stimulation with α6/β4 integrin (Ag)-antibody (Ab) immune complexes (IC). Controls include PMNs (cells) alone, Ab alone, and Ag alone. Results are presented as mean ± standard deviation, with data pooled from three independent experiments and analyzed by Kruskal–Wallis test with Dunn’s multiple comparisons test. * *p* < 0.05; *** *p* < 0.001; **** *p* < 0.0001. (**C**) In cryosections of healthy mouse skin, IgG against a recombinant fragment of the NC1 domain of type VII collagen (anti-COL7c IgG; positive control) induces dermal-epidermal separation (arrows) at the dermal-epidermal junction (dotted lines). In contrast, incubation with anti-α6 integrin IgG, anti-β4 integrin IgG, or normal rabbit IgG (NR IgG; negative control) did not result in split formation (*n* = 3). Scale bar, 100 µm.

Next, we employed the ROS release assay to quantify the ability of IC formed by murine α6/β4 integrin fragments and rabbit anti-α6/β4 integrin IgG to induce ROS production from PMNs. All tested IC concentrations significantly elevated ROS release from human PMNs compared to controls, including IgG fractions alone, α6β4 integrin fragments alone, and PMNs alone (Figure 3A,B). In addition, ROS release was significantly higher at an IC concentration of 40 µg/mL compared to 10 µg/mL (Figure 3A,B).

To further assess the pathogenicity of anti-α6 and anti-β4 integrin IgG, we performed an ex vivo cryosection assay, which measures the ability of anti-BMZ antibodies to induce dermal-epidermal separation in cryosections of normal skin following incubation with normal PMNs [22,34]. Unlike anti-Col7 IgG, neither anti-α6 nor anti-β4 integrin IgG fractions induced dermal-epidermal separation in this assay (Figure 3C).

### 3.3. Anti-α6 and Anti-β4 Integrin Antibodies Induce Mild Ocular and Oral Lesions In Vivo

**Figure 4:**
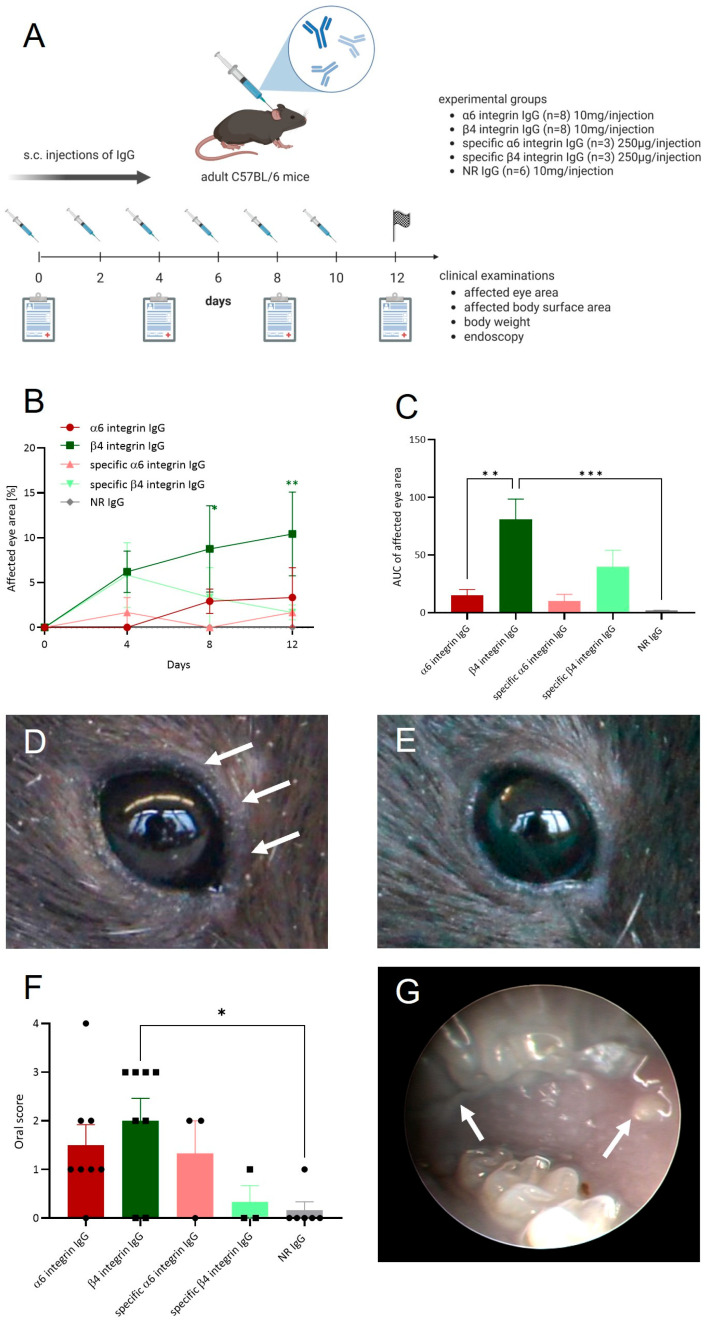
Murine anti-α6 and anti-β4 integrin IgG induce mild clinical phenotypes in vivo. (**A**) Schematic overview of the experimental design. Adult C57BL/6J mice were injected subcutaneously (s.c.) at the neck with either 10 mg of total IgG or 250 µg of specific anti-α6 or anti-β4 integrin IgG every other day (days 0, 2, 4, 6, 8, and 10). Mice were clinically assessed every four days. (**B**,**C**) Mice injected with either total or specific anti-integrin IgG developed palpebral conjunctival swelling. Conjunctival disease progression was significantly more pronounced in mice receiving anti-β4 integrin IgG compared to those injected with anti-α6 integrin IgG. (**B**) The extent of macroscopic eye involvement was quantified as a percentage relative to the total area over time. (**C**) The affected eye area was expressed as the area under the curve (AUC) over 12 days. (**D**,**E**) Representative clinical images. (**D**) Mice injected with specific anti-β4 integrin IgG demonstrated a trend towards increased palpebral conjunctival involvement (arrows). (**E**) Representative image of the eye from mice injected with normal rabbit IgG (NR IgG; negative control). (**F**) Endoscopic scoring on day 12 revealed mild mucosal involvement in anti-β4 integrin IgG-injected mice. (**G**) Representative endoscopic image of the buccal mucosa from anti-β4 integrin IgG-injected mice on day 12 showing mucosal lesions (arrows). Results are presented as mean ± SEM. Data were analyzed by two-way or one-way ANOVA and with Dunnett’s multiple comparison test. * *p* < 0.05, ** *p* < 0.01, *** *p* < 0.001.

To investigate the in vivo effects of anti-α6 and anti-β4 integrin IgG, adult C57BL/6J mice were injected s.c. at the neck every other day for 10 days. Mice received either total anti-α6 or anti-β4 integrin IgG (10 mg per injection; *n* = 8 per group) or anti-α6 and anti-β4 integrin IgG specifically purified using recombinant integrin fragments (250 µg per injection, *n* = 3 per group; Figure 4A).

Palpebral conjunctival swelling was observed in animals receiving total or specific anti-β4 integrin IgG, but not in mice injected with NR IgG (*n* = 3 per group) (Figure 4B–E). Over the 12-day period, palpebral swelling was significantly greater in the total and specific anti-β4 integrin IgG groups compared to the NR IgG group, reaching significance on days 8 and 12 in mice receiving total anti-β4 integrin IgG (Figure 4B,C). In contrast, no conjunctival swelling was observed in mice treated with anti-α6 integrin IgG (Figure 4B,C,E).

On the final day of the study, endoscopic evaluation of the oral cavity revealed mild oral lesions in all experimental groups, except for the control mice (Figure 4F,G). Mice receiving total anti-β4 integrin IgG and total anti-α6 integrin IgG exhibited significantly more oral involvement compared to those injected with NR IgG (Figure 4F). No differences in the extent of oral lesions were observed in mice receiving specific anti-α6/β4 integrin IgG fractions (Figure 4F).

No obvious skin lesions were observed in any of the experimental groups (Figure S1A), and body weight remained stable across all groups throughout the study (Figure S1B). The absence of body weight loss further underscores the relatively mild nature of the oral lesions induced.

### 3.4. Anti-α6 and Anti-β4 Integrin IgG Induce Inflammatory Infiltrates but No Subepithelial Splitting

**Figure 5:**
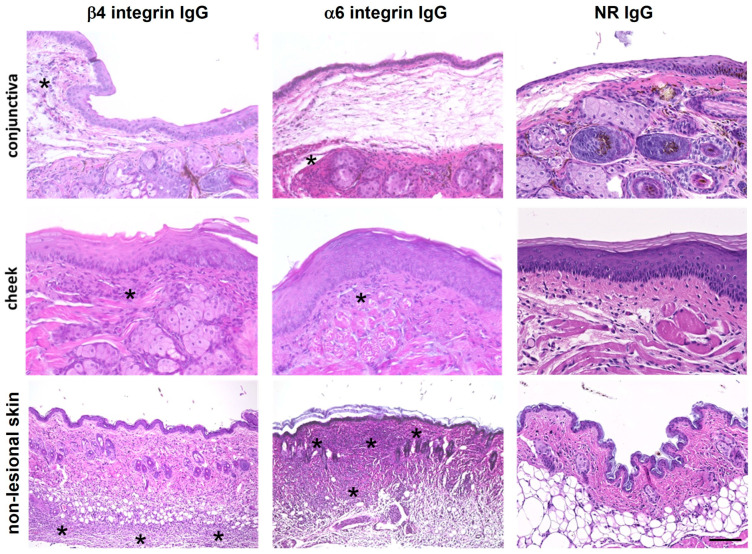
Anti-α6 and anti-β4 integrin IgG induce inflammatory infiltrates in mucosal tissues and skin of injected mice. Tissue biopsies were obtained from mice on day 12 of the experiment. H&E-stained sections from the conjunctiva, buccal mucosa (cheek), and non-lesional skin of mice injected with 10 mg of anti-α6 integrin IgG, anti-β4 integrin IgG, or normal rabbit (NR) IgG (negative control) are shown. Asterisks mark areas of immune cell infiltration. Injection of anti-α6 and anti-β4 integrin IgG induced inflammatory infiltrates with varying intensities: mild in the conjunctiva, moderate in the buccal mucosa, and severe in the non-lesional skin. No inflammatory infiltrates were evident in NR IgG-treated controls. Scale bar, 100 μm.

Histopathological analysis of biopsies from the conjunctiva, buccal mucosa, hard palate, and skin adjacent to the IgG injection sites revealed an inflammatory infiltrate in all tissues from mice injected with total anti-α6 or anti-β4 integrin IgG (Figure 5 and Figure S2). The inflammatory infiltrate was most prominent directly below the epithelium in conjunctival and buccal biopsies. In skin biopsies, inflammatory infiltration was observed in both the upper and lower dermis. No inflammatory infiltrates were detected in mice injected with NR IgG. Additionally, no subepithelial or subepidermal splitting was observed in any of the tissue sections (Figure 5), except for discrete split formation in hard palate biopsies of two anti-α6 integrin IgG-injected mice (Figure S2).

### 3.5. Limited Complement Activation Reflects the Mild Clinical Phenotype of Anti-α6 and ASnti-β4 Integrin IgG-Injected Mice

**Figure 6:**
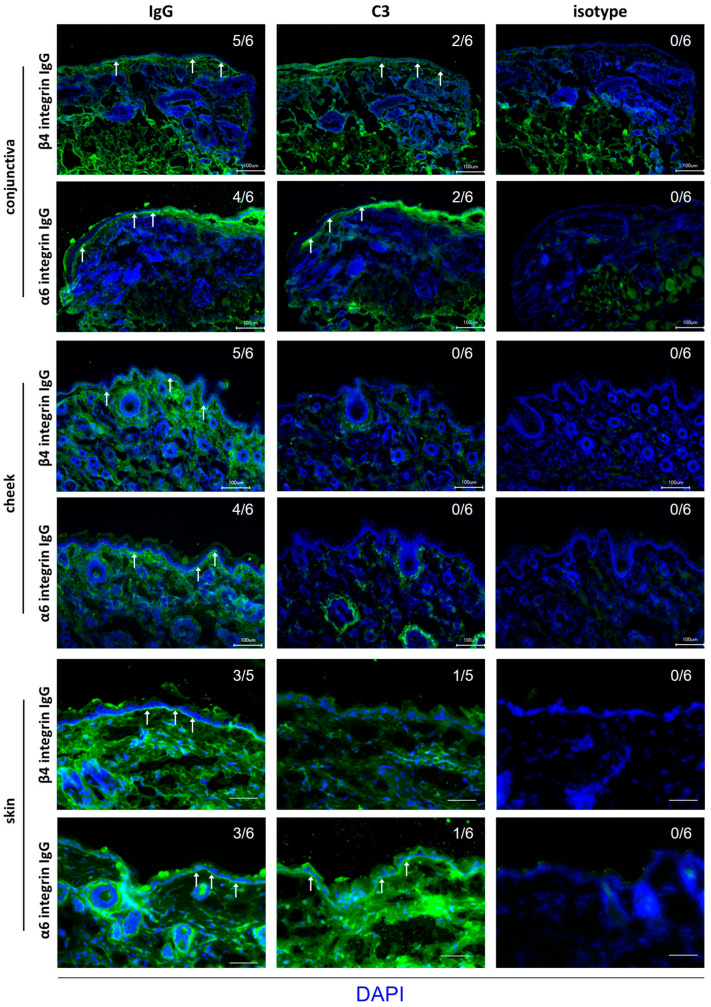
Anti-α6 and anti-β4 integrin IgG bind along the basement membrane zone (BMZ) of mucosal and skin tissues. Direct immunofluorescence revealed linear IgG deposition (arrows) along the BMZ of conjunctiva (5/6 and 4/6), skin (3/5 and 3/6), and buccal mucosa (5/6 and 4/6) biopsies from mice injected with anti-β4 or anti-α6 integrin IgG, respectively. Linear C3 deposition (arrows) was also detected in the conjunctiva (2/6 and 2/6) and skin (1/5 and 1/6) of anti-β4 or anti-α6 integrin IgG-injected animals, respectively. Representative images with positive staining are shown. Notably, no C3 deposition was detected in the buccal mucosa of either experimental group. Sections stained with an isotype antibody served as negative controls. Scale bars, 100 μm.

Direct IF microscopy was performed in biopsies from the conjunctiva, buccal mucosa, and skin adjacent to the IgG injection sites of all three experimental groups. In most sections from mice injected with anti-α6 and anti-β4 integrin IgG, linear IgG deposition was observed along the BMZ (Figure 6). In contrast, linear C3 binding was seen only in a few conjunctival and skin biopsies and was absent in buccal mucosa samples (Figure 6).

**Figure 7:**
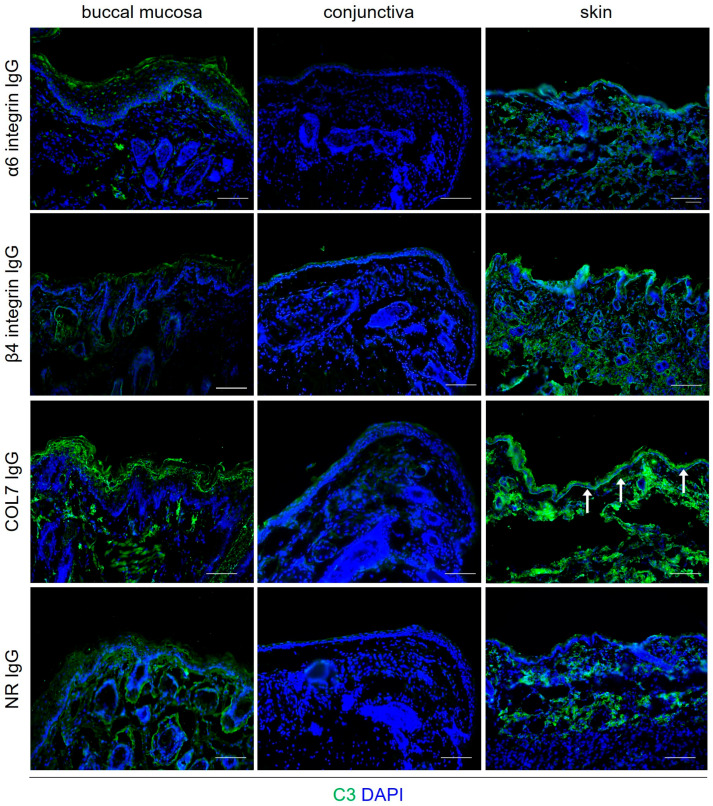
Anti-α6 and anti-β4 integrin IgG fail to fix complement along the BMZ in vitro. Representative indirect immunofluorescence microscopy images of murine buccal mucosa, conjunctiva, and skin biopsies are shown. Following incubation of sections with IgG, fresh hirudin plasma was added as a complement source. Complement activation was assessed using anti-C3 staining. Robust linear C3 deposition (arrows) along the BMZ was observed in normal murine skin treated with IgG against the recombinant NC1 domain of collagen type VII (anti-COL7 IgG, positive control). In contrast, no C3 deposition was detected in tissues treated with anti-α6 integrin IgG or anti-β4 integrin IgG, indicating their inability to activate complement in vitro. Sections incubated with normal rabbit (NR) IgG (negative control) served as negative controls. Nuclei were counterstained with DAPI (blue). Scale bars, 100 μm.

To further corroborate these findings, a complement fixation test was performed to evaluate the ability of anti-α6 and anti-β4 IgG to activate tissue-bound complement in vitro. Tissue sections from the palpebral conjunctiva, buccal mucosa, and skin of healthy adult mice were incubated with purified anti-β4 and anti-α6 integrin IgG, as well as with anti-type VII collagen (anti-Col7 IgG; positive control) and NR IgG (negative control). Across three independent experiments, no C3 deposition was detected along the BMZ in conjunctiva, oral mucosa, and skin sections incubated with anti-α6 or anti-β4 integrin IgG (Figure 7). As expected, anti-Col7 IgG led to robust C3 deposition along the BMZ in skin sections, whereas no C3 deposition was observed with NR IgG (Figure 7).

Since glycosylation of antibodies was shown to contribute to complement activation in another pemphigoid mouse model, we performed glycosylation profiling of anti-α6 and anti-β4 integrin IgG alongside NR IgG (negative control) and anti-mCOL7 IgG (positive control) [35]. The peak composition in all samples was similar, with no major differences (Figure S3). When further analyzing 6 selected peaks for their proposed glycan structures, none of them differed remarkably in their ratios between proposed structures across samples and the negative control.

## 4. Discussion

Integrin α6β4 has been identified as a target antigen in individual patients with MMP [10,11,12,13,17,36,37,38]. However, studies investigating autoantibody reactivity of MMP patients to α6 and β4 integrin have yielded variable results, likely due to differences in methodology and patient populations. For example, β4 integrin reactivity was detected in 6 out of 40 MMP patients in one study and in 26 out of 124 MMP patients in another, with additional reactivity to BP180 [39,40]. While earlier research suggested a correlation between anti-α6 reactivity and anti-β4 integrin reactivity and ocular lesions [13,15,41], more recent studies have not consistently confirmed this observation [10,39]. In an Italian cohort of 40 MMP patients, only 6 exhibited reactivity against β4 integrin, with four displaying exclusively oral involvement without ocular pathology [39]. In contrast, a Japanese cohort of 43 patients with pure ocular MMP showed IgG reactivity to β4 integrin in 63% (27/43) and IgA reactivity in 7% (3/43) of cases, as determined by immunoblotting using a hemidesmosome-rich fraction [10]. However, these results were not consistently replicated using indirect IF microscopy. Notably, none of the pure ocular MMP sera reacted with autoantigens in immunoblots prepared from normal human epidermal or dermal extracts or purified human laminin 332. Overall, 42% of cases showed reactivity to the cytoplasmic domain of β4 integrin, while 19% exhibited reactivity to the α6 integrin ectodomain, previously associated with oral pemphigoid. Collectively, these findings highlight the diverse autoantigen specificity in MMP and underscore the importance of analytical methods used to detect autoantibodies.

Several in vitro studies have explored the pathogenic effects of anti-α6/β4 integrin antibodies. Chan et al. demonstrated that both ocular MMP sera and affinity-purified IgG induced BMZ separation in a cultured human bulbar conjunctiva model, even in the absence of inflammatory cells [15]. Similarly, an in vitro organ culture model showed that anti-BMZ autoantibodies present in MMP patient sera can cause BMZ separation in normal human buccal mucosa, closely resembling the histopathological features of MMP [16]. Furthermore, oral pemphigoid sera and anti-α6 integrin antibodies induced BMZ separation in normal human buccal mucosa following a 48 h-treatment, but not in normal human skin or conjunctiva [17]. In another study, normal human buccal mucosa cultured with MMP sera and rabbit antibodies targeting intracellular domains of β4 integrin exhibited histologically identical BMZ separation [17]. More recently, a blocking antibody (P3H9-2) directed against the G2 domain of laminin, which interacts with integrin α6β4 and integrin α3β1, was shown to induce blistering through keratinocyte reprogramming [42]. Collectively, these findings suggest that anti-integrin antibodies, particularly those with blocking capabilities, may exert similar pathological effects at the BMZ.

Our in vitro results demonstrated that both anti-α6 and anti-β4 integrin IgG stimulated CXCL2 release from murine C5N keratinocytes. CXCL2, the murine functional homologue of human IL-8, is a potent chemoattractant that facilitates the recruitment of neutrophils and monocytes to sites of inflammation [43,44]. In line with this, OMMP patients exhibit significantly elevated IL-8 levels in tears, as well as increased conjunctival expression of IL-8 and CXCR1, compared with healthy controls [45]. It should be noted, however, that C5N keratinocytes are of epidermal origin, not ocular-derived, and may not fully recapitulate the corneal epithelial biology of ocular surface epithelia.

Neutrophils serve as primary effector cells in MMP pathogenesis, contributing to tissue damage through protease release, ROS production, and chemokine-mediated recruitment [2]. In addition, monocytes, upon migrating to inflamed tissues in MMP, differentiate into macrophages. Tissue-resident macrophages can be activated to further recruit neutrophils by secreting chemoattractants such as CXCL1 and CXCL2. Additionally, they prolong neutrophil lifespan by producing granulocyte-macrophage colony-stimulating factor (GM-CSF), G-CSF, and TNF-α [46]. As such, the induction of CXCL2 release from cultured keratinocytes upon stimulation with anti-α6 and anti-β4 integrin IgG may indicate a role of this chemokine in local neutrophil recruitment, potentially contributing to blister formation in MMP [47]. Consistent with this concept, immune complexes formed by anti-integrin autoantibodies induced ROS release from human PMNs, further implicating them in MMP-associated inflammatory cascades. Despite these robust inflammatory responses, neither anti-α6 nor anti-β4 integrin IgG induced dermal-epidermal separation in an ex vivo cryosection assay. While this assay is well-established for evaluating skin blistering, it may not fully capture pathogenic mechanisms operative in ocular or oral mucosa. Unfortunately, a validated ocular cryosection model is currently not available.

A previous study reported that antibodies targeting an intracellular epitope of integrin β4 caused subepidermal cutaneous blistering in neonatal BALB/c mice, although ocular or oral lesions were not described [13]. To further assess the in vivo pathogenicity of α6β4 integrin subunits, rabbit IgG against murine α6 or β4 integrin fragments was administered to adult C57BL/6J mice. Repeated injections of anti-β4 integrin IgG led to conjunctival swelling, partially mimicking ocular manifestations of MMP. Interestingly, both anti-α6 and anti-β4 integrin IgG induced mild oral lesions. These observations align with previous reports [11,12] and challenging the proposed dichotomic association of anti-α6 integrin antibodies with oral lesions and anti-β4 integrin IgG with ocular involvement. However, the clinical severity in this mouse model was relatively mild, likely due to limited complement activation as indicated by only minimal C3 deposition at the BMZ by direct IF. In contrast, C3 deposition at the BMZ is recognized as a diagnostic marker in MMP patients, and C5aR1 activation was shown to be essential for lesion formation in anti-laminin 332 MMP [5,20]. In line, the complement fixation assay confirmed the absence of extensive C3 activation in murine skin and mucosal tissues treated with anti-α6 or anti-β4 integrin IgG. These results suggest that, although integrin autoantibodies can initiate local immune responses, they may not activate the complement cascade sufficiently to cause the overt microscopic and macroscopic blistering characteristic of MMP, thereby explaining the mild lesions observed in vivo. However, the precise role of the complement system in MMP pathophysiology remains an area of active investigation, with growing interest in its potential as a therapeutic target [48,49,50].

Given the established role of glycosylation in shaping complement activation [35,51], we examined the glycosylation patterns of anti-α6/β4 integrin IgG. No significant differences were detected in the glycosylation of anti-α6 or anti-β4 integrin antibodies compared to anti-Col7 IgG. Since anti-Col7 IgG activated C3 on mouse tissues in vitro, these data argue against the hypothesis that altered IgG glycosylation is related to scarce complement activation observed with anti-integrin antibodies.

One limitation of this study is the use of short *E. coli*-expressed integrin fragments. Although these fragments target major pathogenic MMP epitopes and bind specifically to murine skin, full-length α6/β4 ectodomains produced in mammalian or insect cells would better preserve native conformation, disulfide bonding, and N-glycosylation, potentially generating more pathogenic, conformation-dependent antibodies. Eukaryotic full-length models are therefore essential to faithfully recapitulate human MMP pathogenicity. Another limitation of this study is that a complete demonstration of the role of complement in anti-integrin autoantibody-mediated pathology could not be provided. In particular, additional analyses of other complement components, such as C1q (e.g., assessment of rabbit IgG binding), were not performed. Furthermore, the detailed molecular mechanisms underlying the binding of anti-β4 integrin IgG to its cytoplasmic epitope were not investigated.

In conclusion, this study provides new insights into the pathogenic potential of anti-α6 and anti-β4 integrin autoantibodies in MMP. While these antibodies can trigger local inflammation and tissue-specific immune responses, their inability to cause significant tissue damage is possibly due to only minimal complement activation. At present, convincing evidence that integrin-specific autoantibodies alone are sufficient to induce clinically overt MMP is still lacking. Future studies should therefore focus on identifying potential co-pathogenic autoantibodies, as well as the role of complement and additional immune effector pathways in anti-α6/β4 integrin-associated MMP.

## 5. Conclusions

Taken together, our findings demonstrate that autoantibodies directed against integrin α6 and β4 possess pathogenic potential by inducing proinflammatory responses, including CXCL2 release from keratinocytes and ROS generation by neutrophils, thereby contributing to local immune activation in MMP. However, despite these inflammatory effects, these antibodies elicited only mild oral and conjunctival lesions in vivo, accompanied by minimal C3 deposition and limited complement activation. These results suggest that integrin-specific autoantibodies alone are insufficient to cause the full spectrum of MMP pathology, and that robust complement activity and/or additional co-pathogenic autoantibodies may be required for overt blister formation. Collectively, this study expands current understanding of α6β4 integrin-directed autoimmunity and highlights the need to further elucidate the interplay between antibody specificity, complement activation, and tissue inflammation in MMP.

## Data Availability

The raw data supporting the conclusions of this article will be made available by the corresponding author, S.E., on request.

## Supplementary Materials

The following supporting information can be downloaded at: https://www.mdpi.com/article/10.3390/biom16030417/s1, Figure S1: No skin phenotype was observed in mice injected with anti-α6 and anti-β4 integrin IgG.; Figure S2: Histology of hard palate tissue.; Figure S3: Glycosylation.

## Abbreviations

The following abbreviations are used in this manuscript:

ABSA	affected body surface area
BMZ	basement membrane zone
CXCL2	C-X-C motif chemokine ligand 2
DMSO	dimethyl sulfoxide
GM-CSF	granulocyte-macrophage colony-stimulating factor
H&E	hematoxylin and eosin
HILIC–SPE	hydrophilic interaction chromatography–solid-phase extraction
IC	immune complexes
IF	immunofluorescence
i.p.	intraperitoneal
MMP	mucous membrane pemphigoid
NR IgG	normal rabbit IgG
PMNs	polymorphonuclear leukocytes
RT	room temperature
ROS	reactive oxygen species
s.c.	subcutaneous
SDS	sodium dodecyl sulphate
