# Supplementary material for: Complement Activation May Drive the Pathogenicity of Anti-α6 and Anti-β4 Integrin Antibodies In Vivo
Source: Biomolecules. 2026 Mar 12;16(3):417. doi: 10.3390/biom16030417 (PMC13024490; doi:10.3390/biom16030417)
Supplement: Supplementary file 1 [file biomolecules-16-00417-s001.zip › biomolecules-3985843-supplementary.pdf]

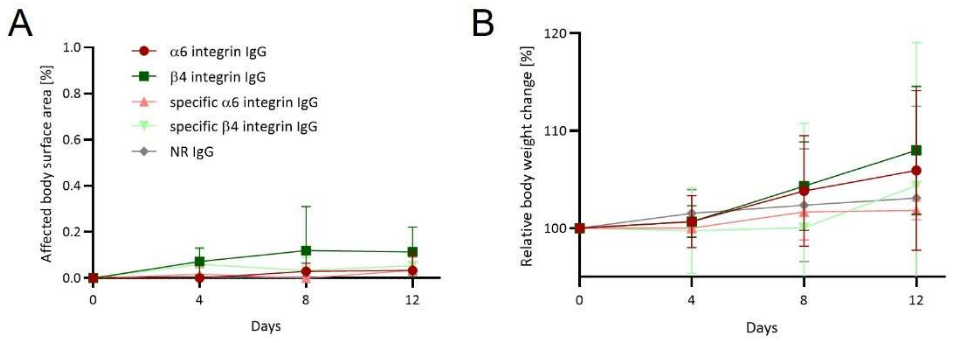

Figure S1. No skin phenotype was observed in mice injected with anti-α6 and anti-β4 integrin IgG. (**A**) The effect of anti-α6 and anti-β4 integrin IgG injections on cutaneous involvement was evaluated by measuring the affected body surface area (ABSA; n=8 mice per group). Neither total IgG nor specific anti-α6 or anti-β4 integrin IgG induced visible skin lesions. (**B**) No significant changes in body weight were detected among the experimental groups. Data are presented as mean ± SEM and were analyzed by two-way ANOVA with the Holm-Sidak multiple comparisons test.

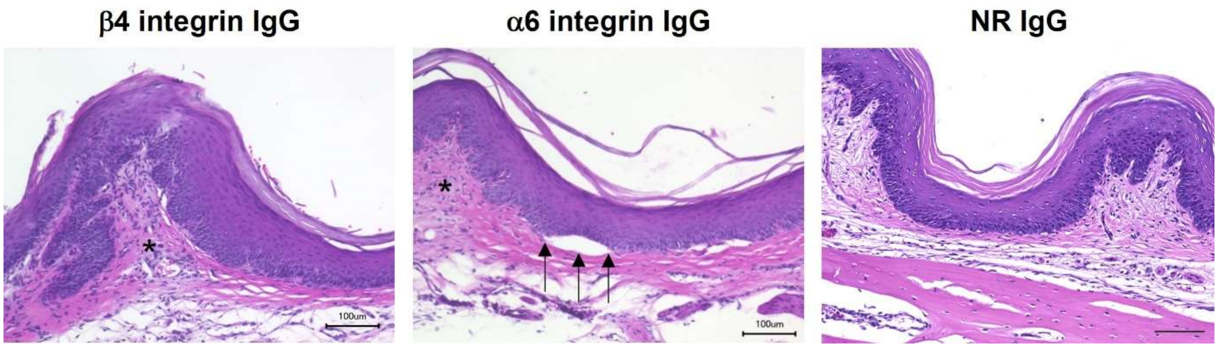

Figure S2. Histology of hard palate tissue. Histological analysis revealed no significant blister formation in the hard palate tissue of mice injected with anti-β4 or anti-*α*6 integrin IgG. Hard palate biopsies were examined for evidence of tissue splitting. Aside from the discrete split formation observed in two mice treated with anti-*α*6 integrin IgG, no blistering was detected in mice receiving anti-β4 integrin IgG, anti-*α*6 integrin IgG, or normal rabbit (NR) IgG. Arrows indicate subepidermal blistering; asterisks denote mild inflammatory cell infiltration. Scale bar, 100 μm.

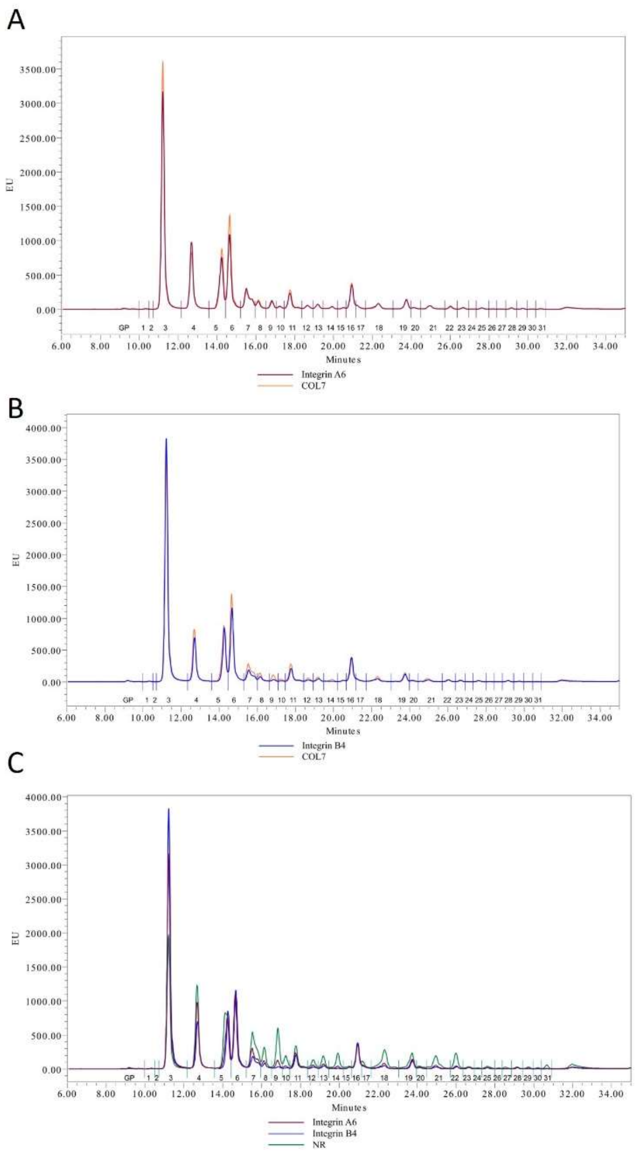

D

% Area	α6 integrin	β4 integrin	COL7	NR
**GP01**	0.13	0.15	0.13	0.05
GP02	0.03	0.03	0.04	0.03
**GP03**	34.14	43.24	36.13	16.29
**GP04**	12.15	9.12	9.72	11.48
GP05	10.25	10.41	10.31	11.6
**GP06**	13.45	14.78	15.59	9.78
GP07	5.97	3.89	5.39	8.22
GP08	1.76	1.33	1.84	3.14
**GP09**	1.73	0.56	1.4	5.61
GP10	0.67	0.28	0.6	2.05
GP11	3.47	3.12	3.77	3.77
GP12	0.96	0.53	0.9	1.7
GP13	1.07	0.78	0.98	2.09
GP14	0.78	0.33	0.72	2.36
GP15	0.37	0.37	0.34	0.54
GP16	4.32	4.67	4.22	3.57
GP17	0.78	0.66	0.8	1.17
GP18	2.08	1.35	1.78	4.78
GP19	2.03	1.69	1.85	2.99
GP20	0.45	0.34	0.48	0.58
GP21	1.21	0.77	1.1	3.05
GP22	0.68	0.48	0.47	2.48
GP23	0.34	0.31	0.37	0.44
GP24	0.08	0.06	0.09	0.16
GP25	0.31	0.26	0.3	0.51
GP26	0.09	0.06	0.08	0.13
GP27	0.09	0.04	0.08	0.25
GP28	0.26	0.25	0.23	0.29
GP29	0.17	0.1	0.14	0.27
GP30	0.06	0.04	0.06	0.15
**GP31**	0.1	0.03	0.1	0.48

Figure S3. Glycosylation. (**A–C**) Chromatograms showing glycosylation profiles of anti-α6 integrin IgG and anti-β4 integrin IgG against anti-COL7 IgG as well as normal rabbit (NR) IgG are depicted. (**D**) Ratios of glycan structures within the identified peaks were comparable across samples, as summarized in the accompanying table. Bold-labeled general peak (GP) intensities were further analyzed by MS/MS N-glycosylation analysis, which revealed no major differences among the tested antibodies (data not shown).
